# Investigating the concept of representation in the neural and psychological sciences

**DOI:** 10.3389/fpsyg.2023.1165622

**Published:** 2023-06-07

**Authors:** Luis H. Favela, Edouard Machery

**Affiliations:** ^1^Department of Philosophy, University of Central Florida, Orlando, FL, United States; ^2^Cognitive Sciences Program, University of Central Florida, Orlando, FL, United States; ^3^Department of History and Philosophy of Science, University of Pittsburgh, Pittsburgh, PA, United States; ^4^Center for Philosophy of Science, University of Pittsburgh, Pittsburgh, PA, United States; ^5^African Centre for Epistemology and Philosophy of Science, University of Johannesburg, Johannesburg, South Africa

**Keywords:** representation, conceptual reform, information, scientific concepts, cognition

## Abstract

The concept of representation is commonly treated as indispensable to research on brains, behavior, and cognition. Nevertheless, systematic evidence about the ways the concept is applied remains scarce. We present the results of an experiment aimed at elucidating what researchers mean by “representation.” Participants were an international group of psychologists, neuroscientists, and philosophers (*N* = 736). Applying elicitation methodology, participants responded to a survey with experimental scenarios aimed at invoking applications of “representation” and five other ways of describing how the brain responds to stimuli. While we find little disciplinary variation in the application of “representation” and other expressions (e.g., “about” and “carry information”), the results suggest that researchers exhibit uncertainty about what sorts of brain activity involve representations or not; they also prefer non-representational, causal characterizations of the brain’s response to stimuli. Potential consequences of these findings are explored, such as reforming or eliminating the concept of representation from use.

## 1. Introduction

The concept of representation is widely applied in research on brains, behavior, and cognition. We call this practice “mainstream representationalism.” Psychologists—especially cognitive psychologists—have historically investigated and explained mental capacities in terms of representations and the computations or operations that process them (e.g., Anderson, 1978; Chomsky, 1980; Fodor, 1981). Examples of this theoretical commitment abound in contemporary work as well, including research on *attitudes* (“attitudes … can be conceptualized as mental representations that determine how we evaluate stimuli”; De Houwer et al., 2021, p. 870), *concept learning* (“[o]ur approach crucially exploits the classic insight that representational *simplicity* is a major determinant of learnability, with learners preferring to infer rules that are concise in their representational system”; Piantadosi et al., 2016, p. 394; italics in original), and *imagery* (“[d]o learners who understand a picture also construct multiple mental representations in their mind”; Schnotz et al., 2021, p. 4). The concept of representation is not only central to research on human mental capacities, but it is also widely applied in research on non-human animals (e.g., “[for] dogs, hearing an object’s verbal label evokes a mental representation of the object”; Dror et al., 2022, p. 8) and on artificial intelligence architectures such as Adaptive Control of Thought-Rational (ACT-R; Anderson et al., 2004), Soar (Laird, 2012), Semantic Pointer Architecture Unified Network (Spaun; Eliasmith et al., 2012), and deep neural networks (e.g., Barrett et al., 2019).

Similarly, the concept of representation plays a central role in the neural sciences. A central theoretical commitment is that brains form representations of the organism’s internal states (e.g., proprioceptive experiences), the external environment (e.g., speed and orientation of visual stimuli), or relational states that cross the internal/external dichotomy (perhaps rewards or beauty). Accordingly, neuroscientists commonly aim at identifying and characterizing these representations in order to answer questions such as the following: what do they represent, what are their vehicles, and how are they used (e.g., Kriegeskorte and Diedrichsen, 2019; Poldrack, 2021)? This is especially true of subdisciplines such as the cognitive neurosciences (e.g., “[w]e usually take for granted the idea that information processing depends on internal representations”; Gazzaniga et al., 2014, p. 74), computational neuroscience (e.g., “[c]learly, the brain must use specific representations and specific algorithms, and it is the goal of computational neuroscience to help find them”; Trappenberg, 2010, p. 12), and sensory neurosciences (e.g., “[t]here is a complete representation of visual space in columns dominated by each eye”; Reid and Usrey, 2013, p. 590).

Following suit, philosophers of psychology and neuroscience have proposed various explications of the concept of representation, sometimes inspired by traditional philosophy of mind (e.g., Von Eckardt, 1995; Ramsey, 2007; Egan, 2014; Shea, 2018), sometimes by work on signaling (e.g., Planer and Godfrey-Smith, 2021), and sometimes by the methods used by neuroscientists to identify neural representations, such as deep learning (e.g., Cao, 2022) and representational similarity analysis (e.g., Roskies, 2021). A minority—but increasingly vocal—group of psychologists (e.g., Richardson et al., 2008), neuroscientists (e.g., Buzsáki, 2019), and philosophers (e.g., Chemero, 2009; Hutto and Myin, 2013) disagree with this mainstream representationalism. They argue that the concept of representation need not be central, or even necessary, to investigate and explain brains, behavior, and cognition.

While the widespread appearance of the concept of representation in the neural and psychological sciences is indubitable, systematic evidence about the ways this concept is applied in these sciences remains scarce (for a rare exception; see Vilarroya, 2017). This article presents four preregistered studies that examine how researchers apply the concept of representation and five other ways of describing how the brain responds to stimuli. This project is in part descriptive: Our main goal was to examine empirically how the concept of representation is used in neural and psychological scientific practice. Additionally, projects such as the current one may also have normative implications. What is at stake is the theoretical status quo concerning the concept of representation, *viz.*, the widespread assumption in the neural and psychological sciences that the concept of representation is understood precisely enough to guide the development of hypotheses, interpretations of experimental data, and explanations. Systematically elucidating what researchers mean by “representation” may draw attention to imprecisions in the concept of representation, which could weaken the strength of conclusions drawn in research that hinges in crucial ways on its meaning. The imprecision of a scientific concept manifests itself in uncertainties concerning what follows from applying it (e.g., “What follows if some brain pattern is a representation?”) and what must be the case for this concept to apply (e.g., “What properties should a brain pattern have to count as a representation?”).

It is important to make clear that the abovementioned issues do not rest on the naive view that a concept can only be appropriately used in scientific research if it is defined by a widely accepted set of necessary and jointly sufficient conditions. Except for formal systems (e.g., logic and mathematics) or a handful of concepts (e.g., the concept of uncle), few concepts can be defined (Machery, 2009), particularly concepts of entities and processes in the natural world. As such, there is no doubt that science progresses without defining all of its terms. Moreover, the absence of definitions can be viewed as an indispensable feature of research when scientists are attempting to characterize novel and interdisciplinary targets of the investigation, as has been the case in the investigations of genes and viruses (e.g., Rheinberger, 2000). Neurophilosopher Patricia Churchland captures well this idea when she writes that to “force precision by grinding out premature definitions enlightens nobody” (Churchland, 1986, p. 346).

While the imprecision of *some* uses of the concept of representation in the neural and psychological sciences can certainly be understood this way, such instances are not what we draw attention to. Consider the following two recent examples from neuroscience. First, in an article on finger movement, the concept of representation is used in contexts such as “different spatial representations,” “low-dimensional representation,” “n members can be represented at time t,” “schematic representation of behavioral mode segmentation,” “the cerebral cortex represents,” and “well-represented in neural state space” (Flint et al., 2020). Second, an article on neural network models of symbolic cognitive processes and dynamical systems uses “representation” in contexts such as “agent’s internal representations of the environment,” “distributed representations,” “feature representation in deep learning,” “holographic reduced representations,” “neurobiological representations (i.e., grid cells),” and “structured symbolic representations” (Voelker et al., 2021). One could reasonably be uncertain about what “representation” means in these instances and what would be required for something to be, for example, a “holographic reduced representation” or “represented in the neural state space.” How does a reader understand if it is reasonable to ask whether a structured symbolic representation (Voelker et al., 2021) can be well-represented in neural state space (Flint et al., 2020)? Granted that these are merely two examples, our goal in this article was to show that they are illustrative of the kinds of imprecision commonly exhibited by uses of “representation” in the psychological and neural sciences. Note that our goal was not to bring to light disagreement about the meaning of “representation” or about variation in how scientists understand this expression across disciplines. On the contrary (as our results demonstrate below), there is little variation in the application of “representation” and related concepts across disciplines.

Moreover, such a project ought to be welcomed by both proponents and critics of mainstream representationalism. Proponents should welcome empirical descriptions of the uses of the concept of representation in order to regiment more tightly how it is used; critics should welcome these descriptions in order to develop more forceful critiques based on a better understanding of the roles the concept of representation plays in the theories of brain, behavior, and cognition that they have long argued does not require representations.

Some handpicked examples, such as those mentioned thus far, are insufficient to provide evidence about how neuroscientists or psychologists use “representation.” All the same, a literature review of the uses of “representation” in even one discipline would be a Herculean task. Thus, how can we empirically assess the current state of neuroscientists’ and psychologists’ understanding of the concept of representation? One option is to utilize what linguists call “elicitation studies” (Greenbaum and Quirk, 1970). Instead of asking scientists to reflect and report on their own concepts or examining the natural occurrences of a given concept (e.g., corpus study), scientists are asked to use the target concept and then the experimenter can make inferences about its content based on subjects’ answers (Machery, 2017; Machery et al., 2019). Inspired by the elicitation-study method, we conducted a survey-based experiment with an international group of neuroscientists, psychologists, and philosophers (*N* = 736). Since the number of respondents was too small to disaggregate the sample along participants’ subdisciplines, each group was analyzed as a combination of subdisciplines. For example, the group of neuroscientists included respondents who identified cellular and molecular neuroscience, cognitive neuroscience, and systems neuroscience as their subdisciplines. We tested the following four preregistered hypotheses:

1. Scientists will not demonstrate statistically significant differences among the concepts selected in the contrasts: representing, carrying information, being about, responding to, processing, and identifying.
2. Scientists will be sensitive to the specificity of content (i.e., high vs. low) but will not be sensitive to its functional integration or the nature of the vehicle (i.e., area vs. population of neurons).
3. On average, scientists will be willing to assign misrepresentations with their average responses falling within the range of either “strongly agree,” “agree,” or “somewhat agree.”
4. In the following three areas: (A) drawing distinctions between concepts; (B) being sensitive to the specificity and functional integration; and (C) being more willing to assign misrepresentation—philosophers will select for more distinctions (A), more sensitivity (B), and more willingness (C), than scientists as a group (i.e., cognitive scientists, psychologists, and neuroscientists).

**Figure 1:**
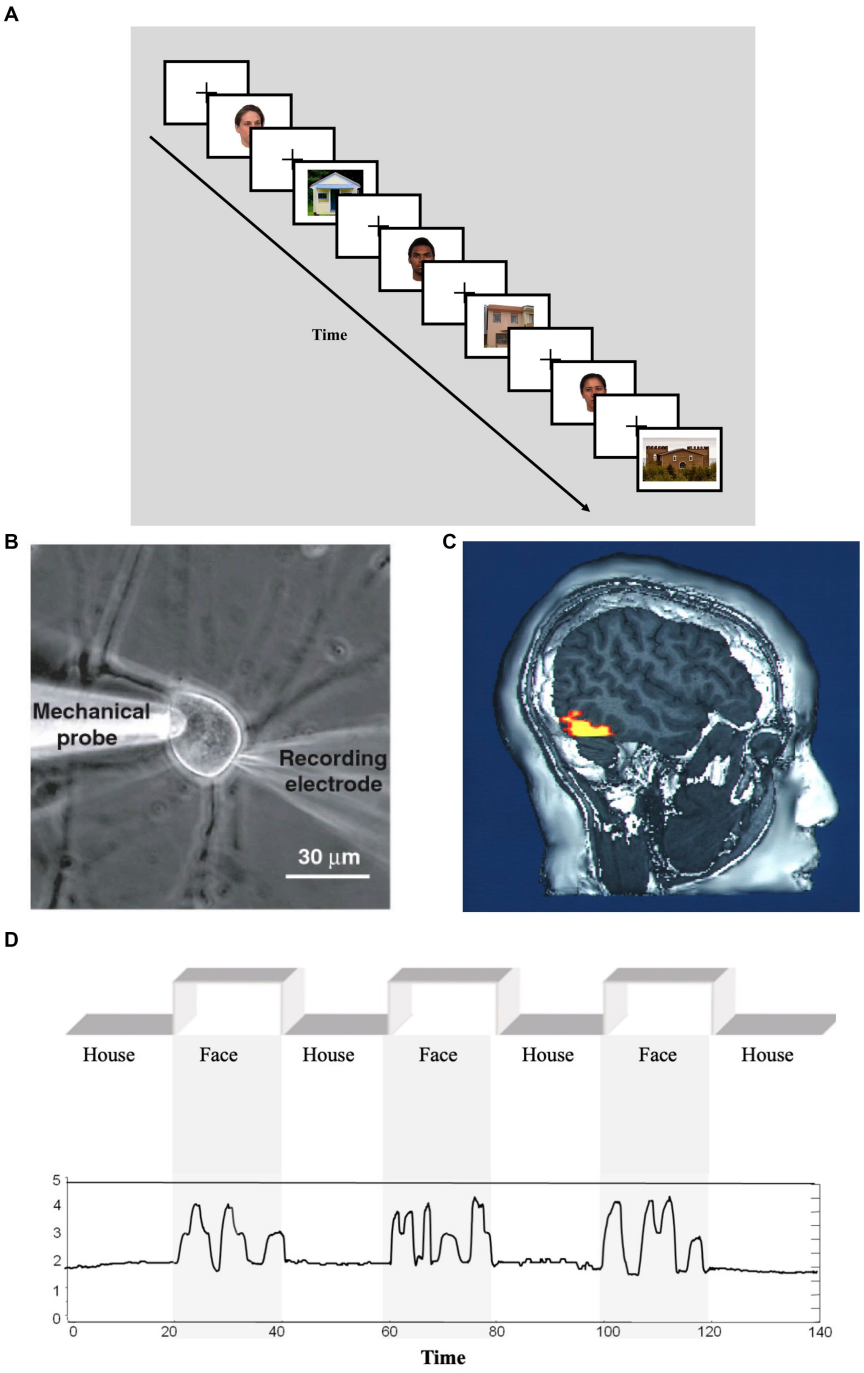
Sample experimental stimuli. The experiment consisted in four studies of similar design, each with a cover story like the one associated with this figure: “In a study published about ten years ago, participants were presented with visual stimuli in a standard block design with alternating images of human faces and houses **(A)**. Data were obtained via a microelectrode **(B)** from single neurons in participants’ fusiform face area **(C)**. An example of the time series data obtained during the task is presented in **(D)**.” Modified and reprinted with permission from Michael J. Tarr, PxHere. CC0 1.0, flickr. CC BY 2.0 **(A)**; (Jia et al., 2013). CC BY 2.0 **(B)**; Public domain **(C)**; (Alkan et al., 2011). CC BY 4.0 **(D)**.

The experiment consisted of four studies with the same basic structure. Participants were given a cover story about a neuroscientific study recording brain response to various stimuli, including faces and artifacts (Figure 1). Participants were then asked to provide a rating on a 7-point scale (from 1: “Strongly agree” to 7: “Strongly disagree”) regarding six questions about whether they would agree to describe the brain’s activity as *representing*, *carrying information*, being *about*, *responding*, *processing*, and *identifying* the stimuli.

These six terms were selected in order to provide participants with ways of describing the brain’s response to stimuli that (1) are used in neuroscience and psychology and (2) are of three different kinds: They describe this response as having an “intentional” component (“representing,” “being about,” and “identifying”), in causal terms (“responding” and “processing”), or as having an information-theoretic nature (“carrying information”). Our goal was to examine whether ways of describing the brain’s response to stimuli that are similar are treated similarly by scientists and to compare scientists’ willingness to use different kinds of descriptions (see Hypothesis 1).

The first term, “representing,” expresses the concept we are investigating. The examples from psychology and neuroscience given above are but a few of the countless instances of researchers using the term in peer-reviewed work. The third term, “about,” or the clause “being about,” describes the brain’s response as intentional. In the current context, being “intentional” means, roughly, standing for something else in the way a sign or a symbol stands for something else. Both describing a response to stimuli as “representing” or as “being about” something assume that this response is correctly understood as intentional (e.g., Baker et al., 2022, p. 945). Aboutness is a central topic in the philosophy of mind (e.g., Yablo, 2014). Responses to stimuli are often described as being about something in psychology, for instance, concerning topics such as language acquisition (Hurford, 2007, p. 173: “[t]he aboutness, or Intentionality, of modern human utterances, derives from the aboutness or Intentionality of pre-linguistic mental representations”) and memory (Klein, 2015, p. 2: “coming from the past does not sanction the inference that the ‘something’ in awareness is about the past”), although the expression is less frequent in neuroscience (except in the context of “information about”). The sixth term, “identifying,” refers to an intentional activity: To identify something (e.g., a face) is more than just responding to some stimuli causally; it involves representing stimuli as something (e.g., as faces). It is sometimes used to describe what the brain does. For instance, Tarr and Gauthier (2000, p. 764) began their influential literature review of the fusiform face area (FFA) by asking “How does the primate visual system process and identify objects?”

The fourth and fifth terms, “responding” and “processing,” differ from the three terms just discussed in describing the brain’s response to stimuli causally, without necessarily implying that this response has an intentional nature. “Responding” is often used in neuroscience, for instance, in research on visual perception (e.g., Barlow et al., 1964; Tarr and Gauthier, 2000, p. 765), with an illustrative phrase being, “retinotopically organized map of neurons responding to parts of visual space” (Ballard, 2015, p. 144). The fifth term, “processing,” is also common (e.g., Tarr and Gauthier, 2000, p. 768: “involved in processing subordinate-level information for all objects, including faces”).

Finally, we use the second term, “carrying information,” to examine whether it would behave more like intentional terms such as “representing,” more like causal terms like “responding,” or in a sui generis manner, perhaps aligned with Shannon and Weaver’s information theory (Shannon and Weaver, 1949/1964). Information-theoretic descriptions of brains’ response to stimuli are very common in neuroscience, including computational neuroscience (e.g., Soh et al., 2018) and cognitive neuroscience (e.g., Ghuman et al., 2014, p. 2: “Recent studies have demonstrated that the FFA activity contains information about individual faces”; Piazza and Eger, 2016, p. 268: “the precise kind of number-related information that is encoded in that part of the brain”).

While these six terms are not exhaustive of all potentially relevant concepts we could have used, for the purposes of our experimental design they capture diverse ways of thinking about the brain’s response to stimuli across the neural and psychological sciences.

The goal of Study 1 was to examine whether neuroscientists, psychologists, and philosophers make any assumptions about the scale at which the vehicles of neural representations, that is, the brain substrates that represent stimuli, are to be found. Participants were randomly assigned to one of two conditions. In the neuron condition, they were told that the response of a neuron was measured by means of a microelectrode (visually represented) when presented with faces; in the population condition, they were told that the response of a neural population (what we refer to in the current study as an “area”^1^; i.e., the fusiform face area (FFA)) was measured by means of functional magnetic resonance imaging (fMRI).

The goal of Study 2 was to examine what kind of relation, if any, must hold between the brain and stimuli for neuroscientists, psychologists, and philosophers to describe it in various terms. Participants were randomly assigned to one of two conditions. In the high specificity condition, a brain area, whose activity was measured by means of fMRI, responded to faces and only to them (it is perfectly specific or selective); in the low specificity condition, it responded to faces but also to houses.

The goal of Study 3 was to examine whether evidence that the brain’s response to stimuli is used by a broader neural network and thus has a function (Cummins, 1975), in addition to a perfect correlation with a stimulus increases neuroscientists, psychologists, and philosophers’ willingness to treat the brain’s response to stimuli in representational terms. It is common to distinguish two kinds of function: teleological vs. “Cummins-style” function (e.g., Millikan, 1989). The teleological function of an object (e.g., an organ or a component of an artifact) explains why this object exists by identifying what it does. In this sense, the function of the heart is to pump blood in the body and the function of glasses is to focus light at the right place of the eye’s lens. Philosophers of biology have often appealed to natural selection and other selective processes (e.g., culture or development) to explain how an object could have a function in this sense (e.g., Millikan, 1989). The Cummins-style function of a part of a system describes how this part causally influences the broader system it is a part of. Study 3 focuses on this notion of function: By embedding the brain area in a broader neural network, our goal was to suggest that the brain causally contributes to a broader system involved in face recognition and, thus, make it clear that it has a particular Cummins-style function. In the mere correlation condition, participants were just given evidence of the brain’s response to the stimuli; in the function condition, the connection between the relevant brain area and a full network was highlighted verbally and by means of two figures.

Finally, the goal of Study 4 was to examine whether neuroscientists, psychologists, and philosophers are willing to describe the brain’s response as erroneous, for example, whether it misrepresents stimuli. Philosophers concur that for a state to count as a representation, misrepresentation must be possible (e.g., Ramsey, 2007, p. 12; Shea, 2018, p. 10). Participants were assigned to a single condition where a brain area that responds to faces happens to also respond, once, to a house.

These four studies focus on characteristics that brain states would have to possess if they are to count as representations. Representations must occur at some scale in brain organization (Study 1); the occurrence of representations must causally depend, in some way, on what they represent (Study 2); representations must be used by downstream processes (Study 3); and representations can be misapplied (Study 4). To have a precise concept of representation is to have a sense of the scale at which representations occur, of the nature of representations’ causal dependence on what they represent, and on the significance of the use of representations, or at least to have some sense for some of these issues. Additionally, a precise concept of representation ought to distinguish cases for which misapplication matters and those for which it does not (Study 4). In what follows, we report the results from each study.

## 2. Methods

### 2.1. Participants

The study was approved by the Institutional Review Boards at the University of Central Florida (IRB STUDY00002612) and the University of Pittsburgh (IRB STUDY20050065). All research was performed in accordance with relevant guidelines/regulations. Hypotheses and data collection methods including the stopping rule, exclusion criteria, and data analytic strategies were preregistered with the Open Science Framework (OSF; https://osf.io/mskwy/; doi: 10.17605/OSF.IO/SARVU).

Two research assistants were tasked to create a database of emails found on the public websites of departments, centers, institutes, and schools at universities around the world. A list of universities in Asia, Australia, Europe, North America, and South America was created, and the research assistants were asked to input the names, emails, and departmental affiliations of cognitive scientists, computer scientists, linguists, neuroscientists, philosophers, and psychologists into a data file. Research assistants were ultimately asked to focus on cognitive scientists, neuroscientists, and psychologists in the United States, setting aside computer scientists and linguists as well as academics from abroad. A total of 14,338 recruitment emails were sent, many of which were blocked by university servers. As was indicated in the preregistration, the study was also advertised on blogs, mailing lists, and social media.

In total, 736 participants completed the study. We excluded participants who reported being younger than 18, who were not graduate students, postdoctoral researchers, professors, or researchers with a doctorate, who either did not respond or gave an incorrect answer to the last question of the survey, “Please tell us what this study was about,” and who provided the same numerical answer to questions in all four scenarios (in line with the preregistration). We also limited our analysis to neuroscientists, psychologists, and philosophers (Table 1), setting aside cognitive scientists in light of the small number of participants who self-identified as such and completed the study (52 before exclusion; a departure from the preregistration).

**Table 1:** Demographic characteristics of neuroscientists, psychologists, and philosophers.

Discipline	*N*	Gender	Age			Highest degree	Location
		W, M, Other	Mean	SD	Range	BA, MA, PhD	United States, United Kingdom, Germany
Neuroscientists	177	39, 59, 2	38.3	13.3	22–77	23, 15, 62	88, 5, 6
Psychologists	159	50, 47, 3	36.7	14.6	21–92	15, 33, 52	87, 2, 3
Philosophers	184	14, 84, 2	43.0	14.4	22–87	2, 24, 74	56, 9, 5

### 2.2. Materials

The recruitment materials included a link to a survey on Qualtrics. Participants were first asked a few demographic questions before being asked to complete successively four studies in a random order (described below). They were then asked several philosophical questions related to representation, computation, and their broader commitments related to the foundations of neuroscience and cognitive science [full survey available at the preregistration site (https://osf.io/mskwy/; doi:10.17605/OSF.IO/SARVU)].

Each of the four studies had the same basic structure. Participants were given a cover story about a neuroscientific study measuring brain response to various stimuli, including faces and artifacts. The first figure represented the basic structure of the experimental design. Additional figures represented the data observed, including a time series. Participants were then asked six questions about whether they would agree to describe the brain’s activity as representing the stimuli, carrying information about the stimuli, being about the stimuli, responding to the stimuli, processing the stimuli, and identifying the stimuli (each on a 7-point scale anchored at “1” with “strongly agree”).

### 2.3. Availability of data and materials, and analyses

The datasets generated and/or analyzed during the current study are available in the Open Science Framework (OSF) repository (https://osf.io/mskwy/; doi:10.17605/OSF.IO/SARVU). All analyses were conducted on R (script available at the preregistration site: https://osf.io/mskwy/; doi: 10.17605/OSF.IO/SARVU). As preregistered, the significance level was set at 0.005 (Benjamin et al., 2018). *p*-values between 0.05 and 0.005 are taken to be suggestive and in need of confirmation. All the analyses were redone with participants who had completed a PhD. The results did not change.

## 3. Results

### 3.1. Mainstream representationalism

**Figure 2:**
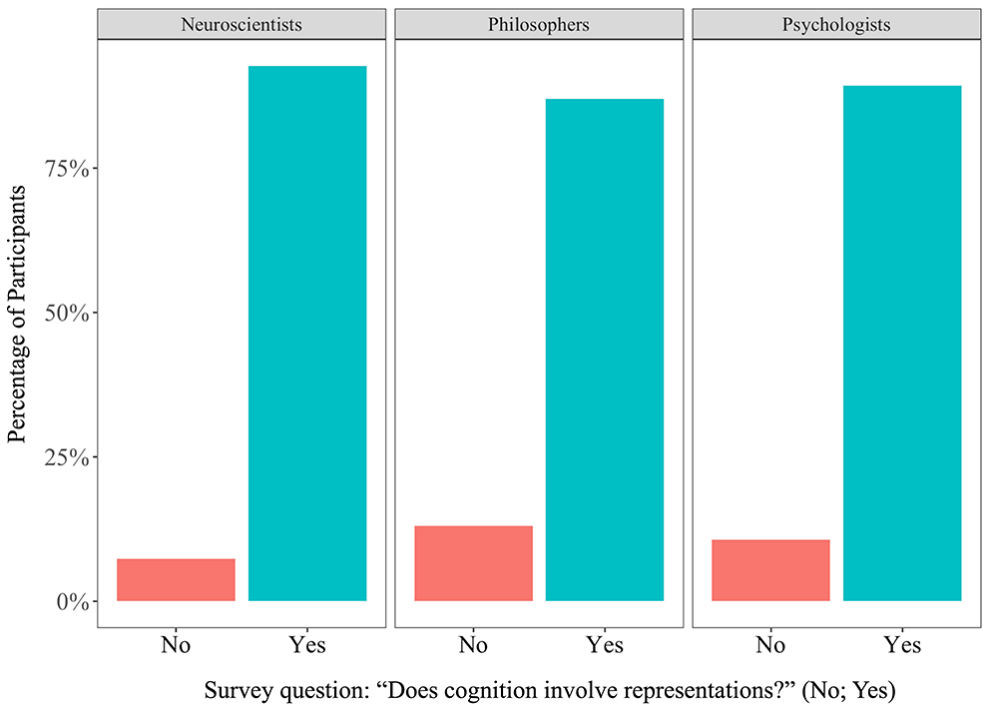
The proportion of “No” and “Yes” answers to the representation question. The overwhelming majority of neuroscientists, philosophers, and psychologists answered “yes” to the question, “Does cognition involve representations?”

Toward the end of the survey, participants were asked five questions aimed at elucidating positions on foundational issues concerning the nature of cognition. We begin by reporting the results from a question probing their commitment to mainstream representationalism: “Does cognition involve representations? Yes or no.” We claimed at the start that mainstream representationalism, i.e., mental capacities involve computations acting on representations and that brains represent stimuli—is widely accepted as being necessary to investigate and explain brains, behavior, and cognition. As expected, a very large majority of participants answered this question positively for the three disciplines of interest (Figure 2). It thus appears that mainstream representationalism is embraced by a large majority of psychologists, neuroscientists, and philosophers.

### 3.2. Study 1: vehicles of representations

**Figure 3:**
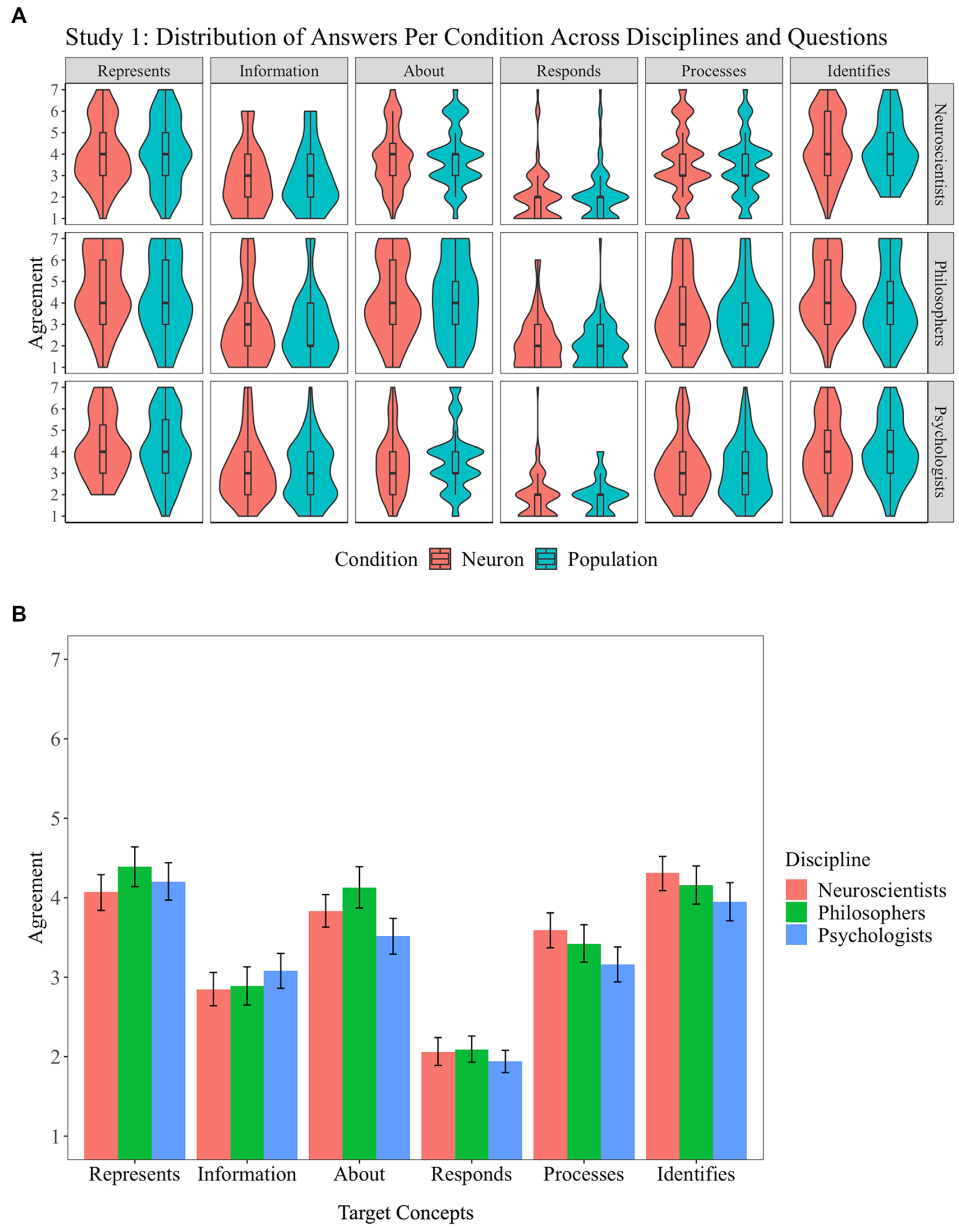
Study 1: Vehicles of representations. Distribution of answers for Study 1 (1: “Strongly agree;” 7: “Strongly disagree”) **(A)**. Interaction of question and discipline in Study 1 **(B)**. Error bars correspond to 95% confidence intervals.

The distribution of responses is presented in Figure 3A. A mixed-design analysis of variance (ANOVA) with questions as a within-participant factor (six levels), discipline as a between-participant factor (three levels), and condition as a between-participant factor (two levels) revealed a main effect (Benjamin et al., 2018) of question (*F*(5, 3,083) = 167.8, *p* < 0.001, *η*^2^ = 0.2), a suggestive effect of discipline (*F*(2, 3,083) = 5.0, *p* = 0.007, *η*^2^ = 0.003), and no effect of condition (*F*(1, 3,083) = 3.5, *p* = 0.06, *η*^2^ ≤ 0.001). *Post-hoc* analysis revealed that the suggestive effect observed for discipline is due to a suggestive difference between philosophers and psychologists (*t*(3083) = 3.0, *p* = 0.007, *d* = 0.14); no other comparison reaches the 0.05 level. All *post-hoc* comparisons between the six questions used were significant except for the non-significant comparison between represents and identifies (*t*(3083) = 0.9, *p* = 0.9) and for the suggestive comparison between is about and identifies (*t*(3083) = −3.3, *p* = 0.01). The main effects of discipline and question were qualified by a suggestive two-way interaction (*F*(10, 3,083) = 2.4, *p* = 0.007, *η*^2^ = 0.006; Figure 3B).

In line with this interaction, an exploratory, not-preregistered mixed-design ANOVA with the question as a within-participant factor (six levels) and condition as a between-participant factor (two levels), was conducted for neuroscientists and psychologists separately. For neuroscientists, we observed a main effect of question (*F*(5, 1,049) = 63.5, *p* < 0.001, *η*^2^ = 0.23), no effect of condition, and no interaction (both *p*s > 0.7). All *post-hoc* comparisons were significant except for the non-significant comparisons between represents and is about, represents and identifies, and is about and processes (*p*s > 0.5) and for the suggestive comparisons between represents and processes and is about and identifies (*p*s > 0.01). For psychologists, we observed a main effect of question (*F*(5, 941) = 53.9, *p* < 0.001, *η*^2^ = 0.22), no effect of condition, and no interaction (both *p*s > 0.8). All *post-hoc* comparisons were significant except for the non-significant comparisons between represents and identifies, is about and processes, carries information and processes (all *p*s > 0.15), is about and carries information, and is about and identifies (*p* = 0.055 and 0.057, respectively).

Three main findings emerge from this first study. First, contrary to our first preregistered hypothesis, neuroscientists and psychologists do not treat all of the descriptions of the brain’s response to stimuli identically. The results indicate that neuroscientists and psychologists find acceptable lean, causal characterizations of the brain’s response to stimuli in terms of responding and processing, as well as an information-theoretic characterization (carrying information about). By contrast, they appear uncertain about intentional characterizations. On average, participants chose “neither agree nor disagree” for “representing,” “identifying,” and “being about.” We will come back to this point in the general discussion below. Importantly, neuroscientists’ and psychologists’ overall uncertainty is not the result of a bimodal distribution, which would be indicated by half of the participants willing to strongly agree to use the concept of representation to describe the brain’s response to stimuli and half of them strongly disagreeing. Rather, the distribution is centered around its mean (The same is true of the three other studies.).

Second, the results suggest that it made very little difference to neuroscientists and psychologists whether the vehicle of representation was verbally and pictorially represented as a single neuron or as a brain area. This negative result suggests that neuroscientists and psychologists do not have any expectations about the scale at which representations are to be found in the brain. In other words, they may subscribe to mainstream representationalism, but their concept of representation is not specific enough to dictate what kind of brain structure or pattern at what level of aggregation (neuron, population, and distributed network of populations) would be a representation.

Third, while philosophers were somewhat less likely to agree with our prompts than psychologists, the variation across disciplines was small. This finding suggests that the concept of representation has not specialized in the disciplines we are considering (see Machery et al., 2019 for a discussion of similar results for the concept of innateness in psychology, biology, and linguistics).

### 3.3. Study 2: specificity and representation

**Figure 4:**
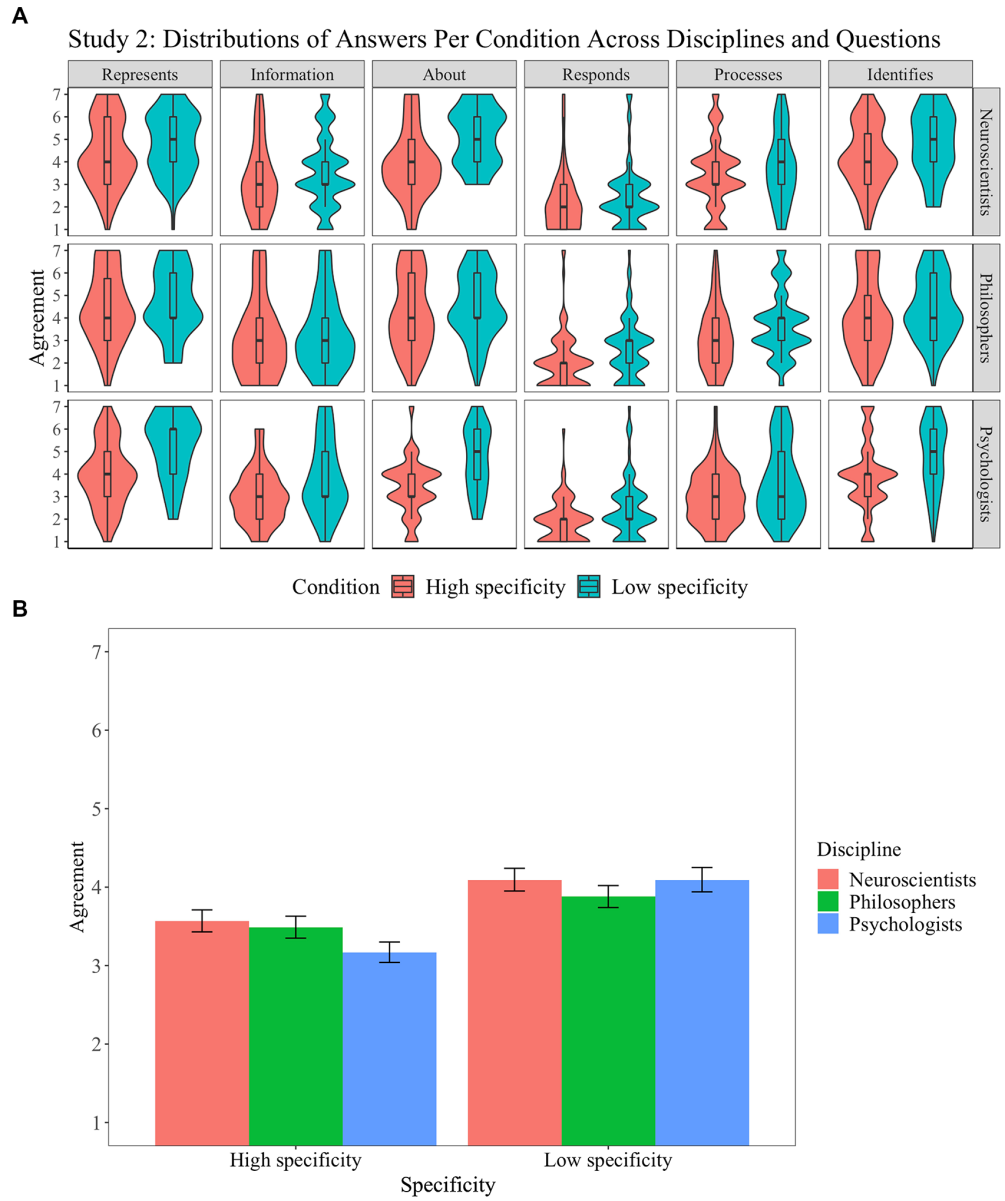
Study 2: Specificity and representation. Distribution of answers for Study 2 (1: “Strongly agree”; 7: “Strongly disagree”) **(A)**. Interaction of condition and discipline in Study 2 **(B)**. Error bars correspond to 95% confidence intervals.

The distribution of responses is presented in Figure 4A. A mixed-design ANOVA with question as a within-participant factor (six levels), discipline as a between-participant factor (three levels), and condition as a between-participant factor (two levels) revealed a main effect of question (*F*(5, 3,083) = 191.4, *p* < 0.001, *η*^2^ = 0.23), a suggestive effect of discipline (*F*(2, 3,083) = 4.9, *p* = 0.007, *η*^2^ = 0.002), and an effect of condition (*F*(1, 3,083) = 131.3, *p* < 0.001, *η*^2^ = 0.03). *Post-hoc* analysis revealed that the suggestive effect observed for discipline is due to a difference between neuroscientists and philosophers (*t*(3083) = 2.6, *p* = 0.03, *d* = 0.1) and neuroscientists and psychologists (*t*(3083) = 3.0, *p* = 0.008, *d* = 0.14). All *post-hoc* comparisons between the six questions used were significant except for the non-significant comparisons between represents and identifies (*t*(3083) = 2.2, *p* = 0.2), between carries information and processes (*t*(3083) = −1.0, *p* = 0.9), and between is about and identifies (*t*(3083) = −0.7, *p* = 0.98) and for the suggestive comparison between represents and is about (*t*(3083) = 3.1, *p* = 0.03). The main effects of discipline and condition were qualified by a two-way interaction (*F*(10, 3,083) = 9.0, *p* < 0.001, *η*^2^ = 0.004): Psychologists are more sensitive to the manipulation of specificity than philosophers and neuroscientists.

In addition, we explored the impact of specificity on representation alone (Figure 4B). For neuroscientists, the impact of specificity on the description of the brain’s response in terms of representation was too small to result in a significant or suggestive effect (*t*(173.07) = −1.90; *p* = 0.059, *d* = 0.1); by contrast, we found a significant effect for psychologists (*t*(155.12) = −5.7; *p* < 0.001, *d* = 0.3).

Two main findings emerge from Study 2. First, as we observed in Study 1, the results indicate preferences by neuroscientists and psychologists for thin, causal descriptions of the brain’s response to stimuli (responds to and processes) and information-theoretic descriptions over intentional descriptions and uncertainty about the latter. Second, specificity matters in describing how the brain responds to stimuli (in line with the preregistered second hypothesis). When one aggregates across ways of describing the brain’s response, neuroscientists, psychologists, and philosophers agree more (although to a different degree) when the brain’s response is maximally sensitive. Turning to the concept of representation, we only found evidence for the significance of specificity for psychologists. It would, thus, seem that psychologists take specificity to be relevant to whether some brain state can count as a representation. However, even perfect specificity does not appear to lead psychologists to express certainty when it comes to describing the brain’s response to stimuli in representational or, more generally, intentional terms.

### 3.4. Study 3: function and representation

**Figure 5:**
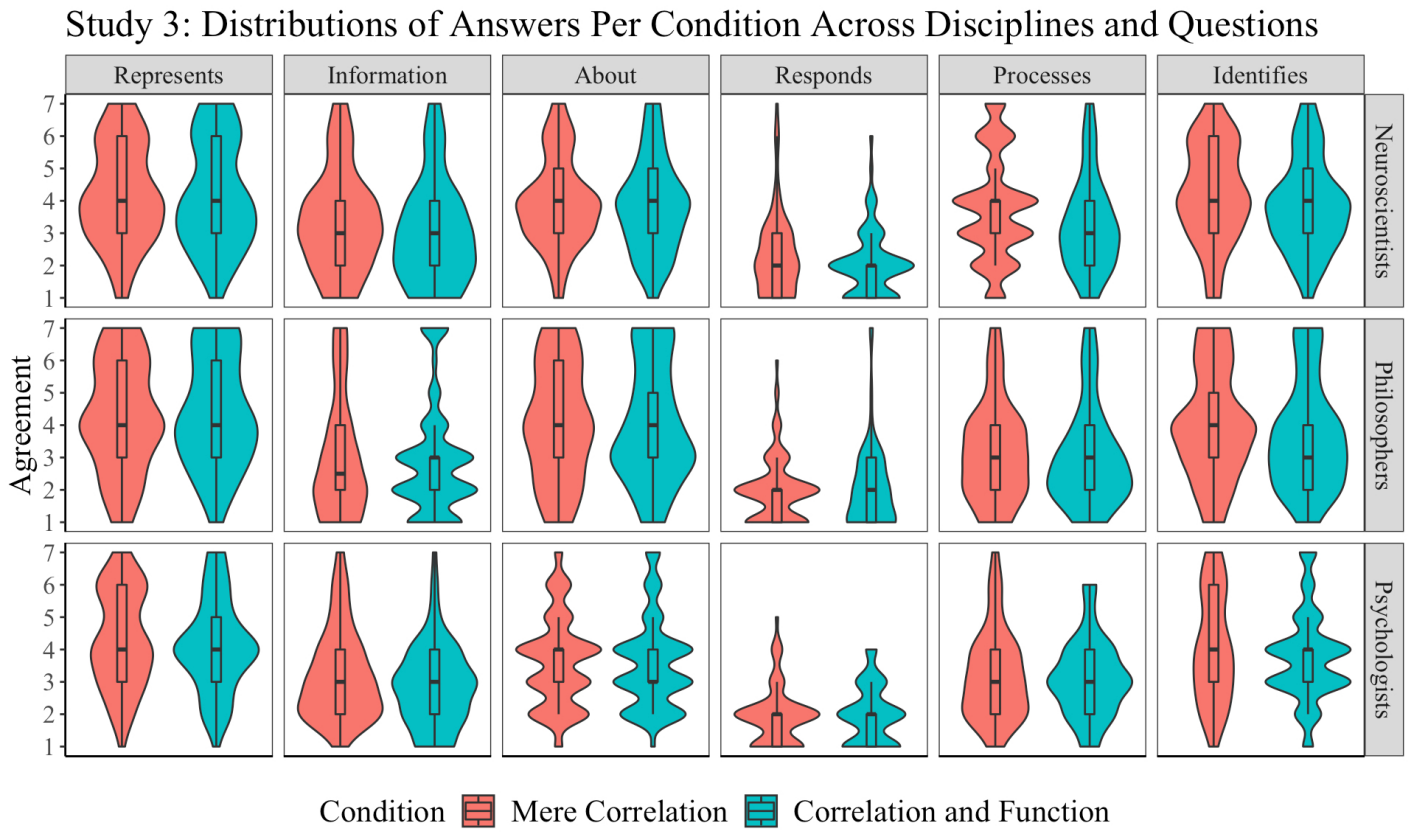
Study 3: Function and representation. Distribution of answers for Study 3 (1: “Strongly agree”; 7: “Strongly disagree”).

The distribution of responses is presented in Figure 5. A mixed-design ANOVA with question as a within-participant factor (six levels), discipline as a between-participant factor (three levels), and condition as a between-participant factor (two levels) revealed a main effect of question (*F*(5, 3,083) = 150.2, *p* < 0.001, *η*^2^ = 0.19), and suggestive effects of discipline (*F*(2, 3,083) = 4.7, *p* = 0.009, *η*^2^ = 0.002) and condition (*F*(1, 3,083) = 6.9, *p* = 0.009, *η*^2^ = 0.002), but no interaction. *Post-hoc* analysis revealed that the suggestive effect observed for discipline is due to a suggestive difference between neuroscientists and psychologists (*t*(3083) = 2.9, *p* = 0.009, *d* = 0.1); no other comparison was significant at the 0.05 level. All *post-hoc* comparisons between the six questions used were significant except for the non-significant comparisons between carries information and processes (*t*(3083) = 2.0, *p* = 0.4) and between is about and identifies (*t*(3083) = −1.0, *p* = 0.9) and for the suggestive comparison between represents and identifies (*t*(3083) = 3.2, *p* = 0.02). To explore the role of function in the assignment of representation, we conducted an ANOVA with question as a within-participant factor (six levels) and condition as a between-participant factor (two levels). No significant or suggestive effect was observed.

Two main findings emerge from Study 3. First, as was found in Studies 1 and 2, the results indicate preferences by neuroscientists and psychologists for thin, causal vocabulary to describe the brain’s response to stimuli and uncertainty about intentional vocabulary. Second, whether or not the brain area’s response to a stimulus is embedded in a larger network, and thus, whether it has a function, influenced how the brain’s response was described, although it did not appear to influence whether it was described in representational terms. When it comes to representation, we found no evidence that having a function matters (in line with the preregistered second hypothesis).

### 3.5. Study 4: misrepresentation

**Figure 6:**
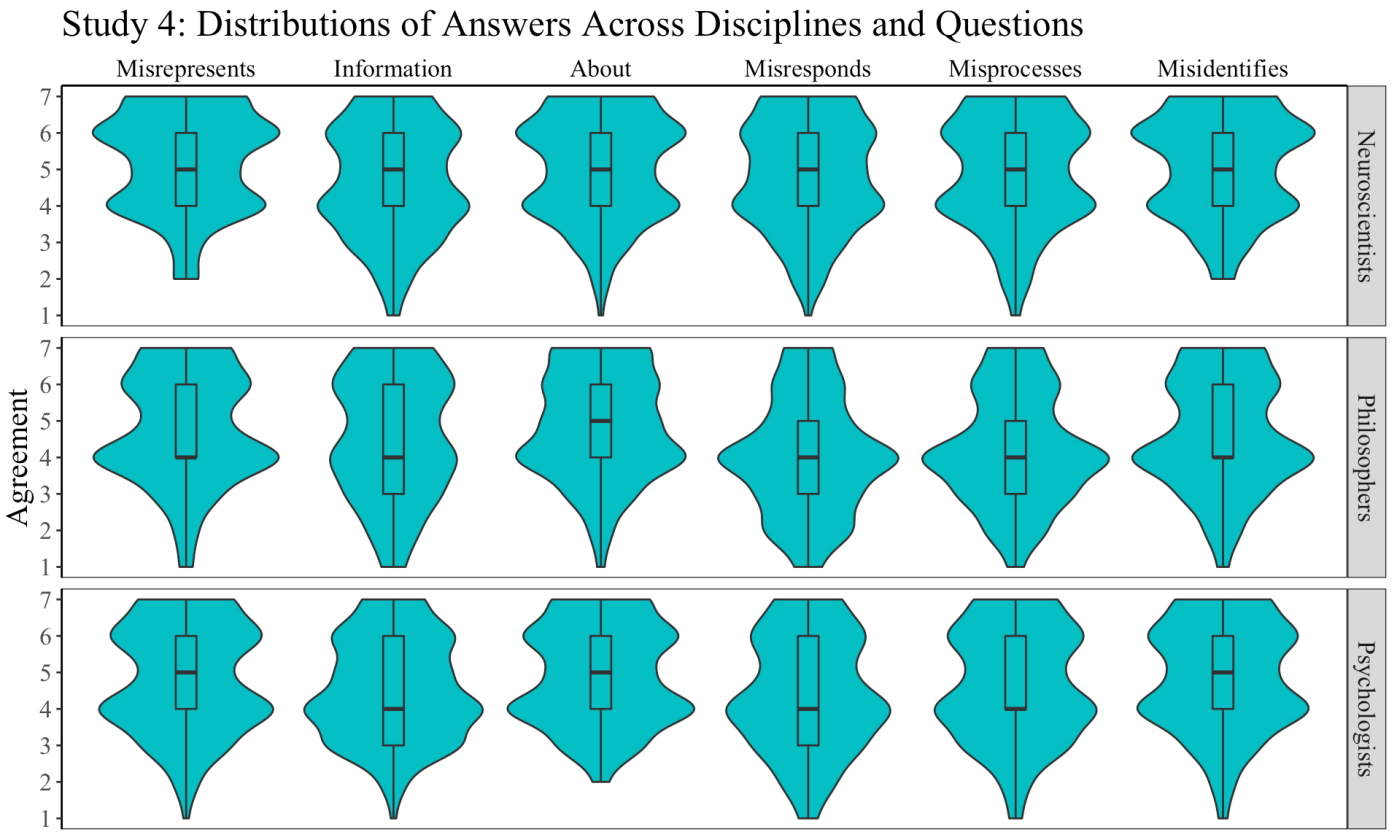
Study 4: Misrepresentation. Distribution of answers for Study 4 (1: “Strongly agree”; 7: “Strongly disagree”).

The distribution of responses is presented in Figure 6. A mixed-design ANOVA with question as a within-participant factor (six levels) and discipline as a between-participant factor (three levels) revealed a main effect of question (*F*(5, 3,101) = 11.5, *p* < 0.001, *η*^2^ = 0.02) and discipline (*F*(2, 3,101) = 26.6, *p* < 0.001, *η*^2^ = 0.02), but no interaction. *Post-hoc* analysis revealed that the effect observed for discipline is due to significant differences between all disciplines, with philosophers being less unwilling to view the brain’s response as erroneous (in line with the fourth preregistered hypothesis). All pairwise comparisons between questions were significant at the 0.005 level, except for represents and is about, represents and identifies, carries information and represents, carries information and processes, is about and identifies, and responds and processes, which were not significant at the 0.05 level, and carries information and identifies and processes and identifies, which were only suggestive (0.01 < *p*s < 0.05). We also found that neuroscientists and psychologists are unwilling to assign misrepresentation (both mean answers significantly higher than the neutral point, “neither agree nor disagree”; *p*s < 0.001).

The main finding to emerge from Study 4 is that neuroscientists, and to a smaller extent psychologists, did not describe the brain’s response to stimuli as erroneous, that is, as failing to do what the brain is meant to do (contrary to the preregistered third hypothesis). In particular, neuroscientists and psychologists appear unwilling to say that it misrepresents something as something else.

## 4. Discussion

Neuroscientists, psychologists, and philosophers commonly embrace the idea that the concept of representation is indispensable to investigating and understanding brains, behavior, and cognition. While neuroscientists, psychologists, and philosophers occasionally differ in their responses to the experimental stimuli used in our four studies, those differences appear to be very small. The concept of representation does not appear to have specialized in different directions as scientific concepts sometimes do when they are used in different disciplines (Hull, 1988; Machery et al., 2019). While our sample size was not large enough to investigate whether the concept of representation varies within disciplines (e.g., between molecular and system neuroscientists), we found no evidence for this since the data were not bimodally distributed. However, further work should address this limitation of the present study and compare neuroscientists across subdisciplines.

Furthermore, having a *precise* concept of representation requires having some sense of what follows from something being a representation (or of what is required for something to count as a representation), including the scale at which it occurs, the way it depends on stimuli, or how it features in downstream processes. Additionally, having a precise concept of representation would facilitate distinguishing representations from other kinds of signs. Despite the centrality of representations for investigating and explaining brains, cognition, and behavior (Figure 2), findings from Studies 1 to 4 suggest that “representation” may express an imprecise concept among researchers.

First, in none of the four studies did neuroscientists and psychologists describe the brain’s response as representing its stimulus. One interpretation of this finding is that participants do not do so because they think this response is not an instance of representation. However, another reason may be their uncertainty about what is required for something to be a representation as illustrated by their neither agreeing nor disagreeing. This pattern is found in other intentional descriptions such as the idea that the brain’s response is about its stimulus or what it identifies as its stimulus. This uncertainty stands in contrast with neuroscientists’ and psychologists’ selections of thinner, causal descriptions, such as responding and processing, to the brain’s response to stimuli. Neuroscientists and psychologists also selected descriptions of the brain’s response in information-theoretic terms, suggesting perhaps that they understand information in more a causal sense than an intentional one.

Second, neuroscientists and psychologists do not appear to have a precise idea about what kind of brain structure or pattern counts as representation. Whether the brain’s response was described at the neuronal (single neuron) or at the population level (what we refer to in the current study as an “area”) made little difference to their answers.

Third, neuroscientists appear not to require the brain’s response to be used in a broader neural network and, thus, to have a function (Cummins, 1975) to count as a representation. They could be indifferent to the role of function for representations either because they endorse a non-functionalist, correlation-based account of representation or because they are uncertain about what is required for something to count as a representation. Their uncertainty in applying the concept of representation noted above suggests that the latter is more likely the case. For psychologists, on the other hand, representation requires specificity, that is, brain states cannot be representations if they occur in response to different types of stimuli. Thus, psychologists’ concept of representation is more precise than neuroscientists’ concept: They appear to endorse a necessary condition for the application of this concept.

These first three points tentatively suggest that psychologists’ and, to an even greater extent, neuroscientists’ concept of representation is imprecise: Psychologists and neuroscientists selected responses indicating uncertainty about what properties a brain pattern must have to count as a representation and what follows from calling a brain pattern a representation. This uncertainty extends to other intentional notions and contrasts with thinner, causal notions.

One of the few things philosophers working on representation agree upon is that representation requires misrepresentation (e.g., Bechtel, 1998; Haugeland, 1998; Ramsey, 2007; Shea, 2018), that is to say, representations can be misapplied; for example, a map can misrepresent the region it is about; we can call a dog a “wolf.” By contrast, a natural sign cannot misrepresent (Dretske, 1988): The smoke produced by the fire carries information about the fire, but it cannot misrepresent it; tree rings carry information about the age of the tree but cannot misrepresent it; and so on. Neuroscientists and psychologists did not select responses that describe the brain’s response as erroneous, including as being a misrepresentation. These choices suggest that their concept of representation may be imprecise to a degree that does not distinguish natural signs and representations.

If, as our results suggest, there is indeed widespread uncertainty in applications of the concept of representation, then such a state of affairs might not be innocuous. They could breed fruitless debates about whether or not some brain part that responds to some stimulus represents it; barring a clearer concept of representation, such debates cannot be resolved. For instance, in the embodied cognition literature, cognitive neuroscientists have provided ample fMRI evidence that at least sometimes (e.g., Kiefer and Pulvermüller, 2012), motor and perceptual areas of the brain are activated when participants retrieve and use concepts, but critics have responded that those activations are incidental: They are not the conceptual representations themselves (e.g., Mahon and Caramazza, 2008). Without greater precision about what it means for a brain pattern to be a representation and some operationalization of the concept of representation, this controversy is unlikely to be resolved. Furthermore, imprecision of the concept of representation could prevent neuroscientists from interpreting some experimental results univocally. fMRI adaptation, multi-voxel pattern analysis (MVPA), representational similarity analysis, and others are supposed to determine what kind of representations the brain produces and where. If the concept of representation at play is genuinely imprecise, then it is hard to say what such methods reveal about the brain.

What is to be done with an imprecise scientific concept such as, possibly, the concept of representation? One approach is that such concepts must be *reformed* or, as philosophers say, “explicated” (Carnap, 1950), “prescriptively analyzed” (Machery, 2017), or “engineered” (Cappelen, 2018). Explication takes an existing concept (either a folk or a scientific concept) and improves it, often in order to use it in philosophical or scientific theorizing. Another approach, well-known in the history and philosophy of science, is to propose to *eliminate* the concept of representation from neuroscience and psychology. In the current context, concepts are eliminated from scientific theorizing when that concept does not refer to anything that actually exists (e.g., the concept of phlogiston in a theory of combustion; Churchland and Churchland, 1998) or enables discourse that is misleading or problematic (e.g., perhaps the concept of qualia in a theory of consciousness; Dennett, 1993; for more on elimination, see, e.g., Churchland (1979) for folk psychological concepts; Griffiths (1997) for the concept of emotion; Griffiths et al. (2009) for the concept of innateness).

Given the extensive use of the concept of representation, it is reasonable to conclude that most neuroscientists and psychologists would strongly prefer the former option, and it is likely that most philosophers of psychology and neuroscience would agree. At the very least, elimination might be impracticable and, at most, quite costly. Still, one might push for the elimination of the concept of representation, an option critics of mainstream representationalism in psychology and neuroscience would prefer. If the concept of representation is to be eliminated, neuroscience would have to put its results, methods, and theories in non-representational terms. While the exact shape of future neuroscience cannot be predicted, it is worth noting that, following our findings that neuroscientists are willing to describe the brain’s response in causal and informational terms, the tools already exist to describe the dynamics of neural processes in non-representational terms (e.g., Izhikevich, 2007; Honey and Sporns, 2008; Shenoy et al., 2013; Sussillo and Barak, 2013; Cunningham and Byron, 2014; Dumas et al., 2014; Zhang et al., 2020; for additional review see Favela, 2020; Favela, 2021).

If reforming is the better option, then doing so for the concept of representation would require specifying to a sufficient degree of precision the characteristics of representation that make something a representation, including its use and its causal dependence on what it represents, and it would distinguish representations from natural signs. Similar to the above discussion regarding the point that both proponents and critics of mainstream representationalism should welcome the current set of studies—albeit, for different purposes—proponents ought to welcome opportunities for widespread reforming of the concept of representation. For example, identifying ways various usages of the concept are misleading or problematic could facilitate a more certain and precise discourse, which would, in turn, enable more fruitful research. While we remain neutral here about which of these two options is preferable, the current study lends support to the idea that the concept of representation requires precisification, work that will benefit our collaborative interests in understanding brains, behavior, and cognition.

## Data availability statement

The datasets analyzed for this study can be found in the Open Science Framework (OSF; https://osf.io/mskwy/; doi: 10.17605/OSF.IO/SARVU).

## Ethics statement

The studies involving human participants were reviewed and approved by Institutional Review Boards at the University of Central Florida (IRB STUDY00002612) and the University of Pittsburgh (IRB STUDY20050065). No consent was required for this study by the Institutional Review Boards.

## Author contributions

LF and EM contributed equally to the manuscript. All authors contributed to the article and approved the submitted version.

## Conflict of interest

The authors declare that the study was conducted in the absence of any commercial or financial relationships that could be construed as a potential conflict of interest.

## Publisher’s note

All claims expressed in this article are solely those of the authors and do not necessarily represent those of their affiliated organizations, or those of the publisher, the editors and the reviewers. Any product that may be evaluated in this article, or claim that may be made by its manufacturer, is not guaranteed or endorsed by the publisher.

## Footnotes

^1^As a reviewer usefully pointed out, this terminology is a simplification: One can identify brain areas by non-neuronal means.
